# Maize germplasm chronosequence shows crop breeding history impacts recruitment of the rhizosphere microbiome

**DOI:** 10.1038/s41396-021-00923-z

**Published:** 2021-03-10

**Authors:** Alonso Favela, Martin O. Bohn, Angela D. Kent

**Affiliations:** 1 Program in Ecology, Evolution, and Conservation Biology, University of Illinois at Urbana-Champaign, Urbana, IL USA; 2 Department of Crop Sciences, University of Illinois at Urbana-Champaign, Urbana, IL USA; 3 Department of Natural Resources and Environmental Sciences, University of Illinois at Urbana-Champaign, Urbana, IL USA

**Keywords:** Microbial ecology, Plant sciences, Agricultural genetics

## Abstract

Recruitment of microorganisms to the rhizosphere varies among plant genotypes, yet an understanding of whether the microbiome can be altered by selection on the host is relatively unknown. Here, we performed a common garden study to characterize recruitment of rhizosphere microbiome, functional groups, for 20 expired Plant Variety Protection Act maize lines spanning a chronosequence of development from 1949 to 1986. This time frame brackets a series of agronomic innovations, namely improvements in breeding and the application of synthetic nitrogenous fertilizers, technologies that define modern industrial agriculture. We assessed the impact of chronological agronomic improvements on recruitment of the rhizosphere microbiome in maize, with emphasis on nitrogen cycling functional groups. In addition, we quantified the microbial genes involved in nitrogen cycling and predicted functional pathways present in the microbiome of each genotype. Both genetic relatednesses of host plant and decade of germplasm development were significant factors in the recruitment of the rhizosphere microbiome. More recently developed germplasm recruited fewer microbial taxa with the genetic capability for sustainable nitrogen provisioning and larger populations of microorganisms that contribute to N losses. This study indicates that the development of high-yielding varieties and agronomic management approaches of industrial agriculture inadvertently modified interactions between maize and its microbiome.

## Introduction

For the past 70 years, modern industrial agriculture has been characterized by technological advances in crop breeding and high input application of nitrogenous fertilizers [1]. Adoption of these agricultural practices has led to increases in global food security, human population growth, and spurred industrialization [1]. While the benefits of these advances for humanity cannot be overstated, they also have far-reaching environmental consequences from the overuse of inorganic nitrogen fertilizers [2]. Currently, more than five million tons of nitrogen fertilizer is applied annually to maize production in the United States [3]. A large fraction of nitrogen fertilizer applied to arable lands is lost through microbial transformations that alter the mobility of nitrogen [4, 5]. Understanding how the plant-associated microbiome has been altered by technological innovations in agriculture could assist in addressing these agronomic problems [6]. Sustaining future agricultural demands will require controlling the detrimental outcomes of the industrial agricultural systems pioneered over the past century.

Assembly of the plant rhizosphere microbiome is driven by plant genetic and evolutionary history [7]. Plant microbiomes play a major role in altering plant resilience, fitness, nutrition, and productivity [6]. Plant hosts selectively filter microorganisms that colonize their rhizosphere [8, 9]. This selective process is heritable across plant cultivars [10, 11], yet the implication of heritability on rhizosphere microbiome function has been relatively unexplored. In modern agriculture, microbiome functions that contribute to crop growth and sustainability have been replaced with agronomic management practices, and the development of modern crop germplasm has been carried out without consideration of the plant microbiome and its functions as an extended phenotype of the crop genome.

Throughout the 20th century, maize breeders have made concerted efforts to optimize yield under a range of agronomic management environments [12, 13]. Since the 1930s, advances in breeding and agricultural management have resulted in steady increases in yield [14]. The introduction of synthetic nitrogen fertilizers to maize began in the 1940s and reached modern levels around the 1980s [15]. During this time, germplasm was selected to produce the greatest grain yield possible under increased nitrogen conditions and plant density [14]. The selection of maize over this period resulted in alterations to plant nitrogen acquisition, root architecture, insect pest interactions, and grain quality [16–19]. In addition, similar selection pressures in other major cereal crops, rice, and wheat, have shown modulation of plant carbon and nitrogen metabolism, resulting in less efficient nitrogen usage [20]. Without selection for maintenance of microbiome functions that contribute to sustainable nutrient acquisition, crop breeding carried out under high nitrogen (N) conditions may have altered how maize interacts with its rhizosphere nitrogen cycling taxa.

**Fig. 1:**
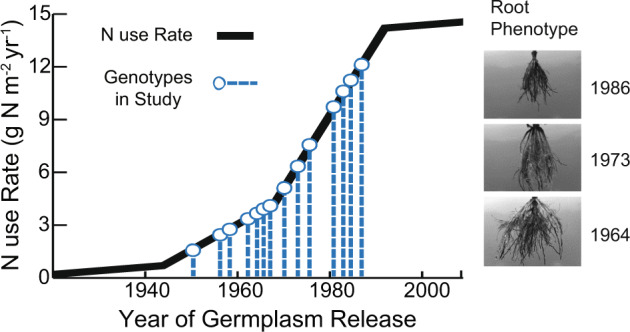
Germplasm chronosequence used in this study mapped on to nitrogen fertilizer use over time. Maize-specific nitrogen use rate was derived from reference [15]. Images highlight the changing root phenotype through time and are from reference [18].

Here, we used a germplasm chronosequence of expired Plant Variety Protection Act maize inbred lines ranging from 1949 to 1986 [18]. These lines act as a genotypic time capsule of the extended phenotype selected by the historic agronomic breeding environment. This time frame was selected as it covers the introduction and increased usage of synthetic N-fertilizers (Figs. 1 and S1, Table S1). The lines used in the study come from two major genetic families: stiff stalk (SS) and non-stiff stalk (NSS). These heterotic groups represent the inbred genetic diversity underlying our modern agricultural elite hybrid varieties.

The goal of this study was to examine if breeding and selection of maize genotypes during the decades of increasing nitrogen application altered how maize germplasm recruits its rhizosphere microbiome, as well as microbiome function. First, we set out to determine if the bacterial and fungal rhizosphere microbiome changed across our chronosequence of maize germplasm. Second, we sought to determine if maize lines developed over the past 50 years difference in their ability to recruit microbial functional groups related to nitrification, denitrification, and nitrogen fixation. Finally, to understand how the metabolic genes of rhizosphere microorganisms change across the germplasm chronosequence, we predicted the metabolic pathways of microbes that responded to the germplasm chronosequence. These results will allow us to determine if crop breeding for yield combined with changing agricultural practices disrupted the interactions between plants and their microbiomes, with potential consequences for nutrient cycling in agroecosystems. If modern breeding has unintentionally transformed the interaction of maize with key functional groups in its microbiome, it must be rewilded to improve agroecosystem sustainability.

## Materials and methods

### Plant genotype selection and greenhouse experiment

Maize seed stocks were obtained from the USDA North Central Regional Plant Introduction Station (Ames, Iowa) and Maize Genetics Cooperation Stock Center (Urbana, Illinois). Twenty inbred lines were selected for comparison: these 20 lines span a breeding period from 1949 to 1986, come from two heterotic genetic groups (SS, NSS), and are adapted for maize production in the U.S. Corn Belt (Table S1). The usage of heterotic groups as a treatment factor was validated using genetic information collected in [21] and available at www.panzea.org. Supplemental Fig. S2 shows that maize genomes cluster based on heterotic genetic grouping. Additional metadata on these lines was acquired from Maize GDB (www.maizegdb.org) and USDA GRIN (www.ars-grin.gov). More information about the history and development of these maize lines is presented in Table S1. Seeds were surface sterilized by soaking for 5 min in 8.25% NaClO, followed by one rinse with sterilized distilled water, a single rinse of 70% ethanol, and three rinses with sterile distilled water. Surface-sterilized seeds were dried on sterile filter paper in a sterile petri dish, then stored at 4 °C overnight before sowing.

Maize lines were grown in greenhouse conditions to isolate the effects of inbred genotype on the microbiome. The planting medium was a combination of live and autoclaved soil mix. The live inoculum soil was collected from agricultural soil located on the Crop Sciences Research and Education Center—South Farms at the University of Illinois at Urbana-Champaign, Urbana, IL. (40°03′31.0″N 88°14′13.4″W). At the time, the soil was out of agricultural rotation (corn-soy) for at least 2 years. Inoculum soil was sieved (2 mm) then added (10%) to a steam pasteurized mix of soil: calcined clay: torpedo sand (1:1:1). An inoculum sample was collected before plant growth to characterize the microbiome before plant treatment. For each genotype, ten replicate classic 600 pots (2 gallons) were sown with three seeds in each. Pots were thinned a week after germination leaving only a single plant per pot for the remainder of the growth. In total, 200 plants were grown. They were placed in a completely randomized design in the greenhouse with 16 h of light and 8 h of darkness. All plants were connected to an irrigation system that fertilized plants twice a week. Plants were fertilized with a liquid nutrient solution, specifically Cal-Mag (N15-P5-K15), at a rate of 150 ppm. Nitrogen was applied as 11.8% nitrate nitrogen, 1.1% ammoniacal nitrogen, and 2.1% urea nitrogen. All plant treatments were maintained under the same fertilizer regime. While a direct comparison of greenhouse fertilization regime to field rates is difficult, the nitrogen level used in this study would be comparable to modern high fertilization levels (Fig. 1).

Implementation of this study in the greenhouse allowed for reduced complexity of environmental factors and homogenization of diverse soil microbiomes typical for a field setting. By reducing random variation, we gained further precision and insight into how different genotypes alter a standardized microbiome.

The roots were harvested 36 days after emergence. Plants were approximately in V4–V5 growth stage with 4–6 fully collared leaves. Plant rhizospheres were harvested by extracting root systems from the soil and shaking vigorously to separate loosely adhering soil. Rhizosphere soil was extracted by placing the root system in a 1-L bottle with 40 mL of sterile distilled water and shaking vigorously for 5 min. The resulting soil slurry was placed into 50 mL centrifuge tubes and lyophilized before DNA extraction using the FastDNA for Soil DNA extraction kit (MPBio, Solon, OH). Rhizosphere samples of all ten replicates for each genotype were harvested for molecular analysis.

### Microbial community amplicon sequencing

For this experiment, we characterized the microbiome and diagnostic functional genes related to transformations that occur in the nitrogen cycle: nitrogen fixation, nitrification, and denitrification. Amplicon sequencing was performed on prokaryotic 16S rRNA genes, fungal ITS2, *amoA*, *nirS*, *nirK*, *nosZ*, *norB*, and *nifH* genes. The Fluidigm access array IFC chip was used to prepare sequencing amplicons. This method allows for the simultaneous amplification of target functional genes using multiple primer sets (Fluidigm, San Francisco, CA). DNA sequencing was performed for bacterial, archaeal, and fungal amplicons using an HiSeq 2500 Sequencing System (Illumina, San Diego, CA). Primer information is provided in supplemental Table S2. Fluidigm amplification and Illumina sequencing were conducted at the Roy J. Carver Biotechnology Center, University of Illinois (Urbana, IL, USA). Fast Length Adjustment of Short reads (FLASH) [22] software was used to merge paired-end sequences from 16S rRNA genes. For functional genes and fungal ITS, only forward read sequences were used. Once reads were merged, they were filtered by quality using the FASTX-Toolkit [23]. Reads that did not have a minimum quality score of 30 across 90% of the bases were removed. Using the FASTX-Toolkit, *nirK* reads were trimmed to their amplicon size of 165 bp.

Once quality preprocessing was performed, FASTQ reads were converted to FASTA format. Using USEARCH-UPARSE version 8.1 [24], sequences were binned into discrete OTUs based on 97% similarity and singleton DNA sequences were removed. Quantitative insights into microbial ecology (QIIME) was used to generate OTU tables for downstream statistical analysis and to assign taxonomic information, this is done with a combination of the UCLUST algorithm and SILVA 138.1 database [24, 25]. Once taxonomy was assigned, chloroplast and mitochondrial OTUs were removed from the dataset. Rarefaction was performed to correct for differential sequencing depth across samples. Functional gene sequences were also assigned using QIIME [26] with the BLAST [27] algorithm and custom gene-specific databases generated from reference sequences obtained from the FunGene repository (http://fungene.cme.msu.edu/) [28]. All OTU tables used in statistical analyses were generated in QIIME. Singleton OTUs were filtered prior to statistical analysis.

The number of raw reads generated from sequencing run reads present after the quality filter, and the rarefaction level of reads per sample for 16S rRNA, ITS, and N-cycling genes are reported in supplemental Table S3. Amplicon sequence data for 16S rRNA genes, fungal ITS2 region, and N-cycling functional genes is available for download on the NCBI SRA database at accession number: P5.

### Quantifying nitrogen cycling functional groups

Quantitative PCR (qPCR) was used to determine the abundance of functional genes in each of the rhizosphere microbial communities. Specific target amplification (STA), explained in [29], was carried out on samples and standards to increase template DNA for amplification. STA and qPCR master mix recipes from [30] were used for all samples. STA product and qPCR master mix were loaded into the Dynamic Array™ microfluidics Fluidigm gene expression chip where amplification and quantification of functional genes were carried out simultaneously (Fluidigm, San Francisco, CA). All samples and standards were analyzed in 12 technical replicates. Fluidigm real-time PCR analysis software version 4.1.3 was used to calculate gene threshold cycles (CT). CT values were converted to gene copy number using gene length and standard curves. All Fluidigm qPCR was conducted at the Roy J. Carver Biotechnology Center (Urbana, IL, USA). The final copy number of each functional gene amplicon was standardized by the ng of template DNA in the qPCR amplification.

### Statistical analysis

The microbial communities were evaluated as separate datasets for each amplicon (16S rRNA, fungal ITS, *nifH*, *nosZ*, *norB*, *nirK*, *nirS*, bacterial *amoA*, and archaeal *amoA*). The relative effect of genotype, heterotic group, genetic relatedness, and decade of germplasm development on the rhizosphere microbiome composition was assessed using permutational analysis of variance (PERMANOVA) with the “adonis” function, from the community ecology R package, “vegan” [31]. To visualize differences from these models, non-metric multidimensional scaling (NMDS) ordinations were created using the R package “phyloseq” and plotted with R package “ggplot2” [32, 33]. Significant differences in functional gene abundance were evaluated using an ANOVA model, and the Tukey’s HSD test from the “stats” package in base R [34]. Correlation between the year of germplasm development and gene abundance was evaluated using “cor.test” and “lm”, packages in base R [34]. Using the “asreml-r” package [35], additional restricted maximum-likelihood mixed-effects models were used to examine the correlation between functional gene abundance and year of germplasm development while controlling for the genetic relatedness between maize inbred lines. TASSEL was used to calculate the pedigree tree, genetic relatedness matrix, and the haplotype diversity (Tajima’s D) across the genome [36, 37].

To control for the variance within individual genotypes when performing our analysis for a decade and heterotic group effects, we used the mean microbiome for each genotype (*n* = 10) (referred to as the genotypic mean microbiome). These mean microbial communities were generated using the “aggregate” function in base R; here this function was used to find the mean of the amplicon data matrix based on the replicates within each genotype.

Modules of microbial taxa responding to the germplasm chronosequence were determined using a weighted correlation network analysis (WGCNA) in R [38]. Prior to WGCNA, amplicon data was transformed using a central log-ratio transformation [39]. PICRUSt2 was used to predict functional pathways present in modules of microbial taxa that change over the germplasm chronosequence [40]. Additional meta-information on predicted PICRUSt2 output was obtained from MetaCyc Database [41]. Graphical representation of workflow present in Supplemental Fig. S3. Similarity percentages analysis (SIMPER) from the “vegan” package was carried out to identify metabolic pathways that were significantly altered in representation across the germplasm chronosequence [42]. Correlation between the year of germplasm release and pathway abundance was evaluated using “cor.test” and “lm” packages in R.

## Results

In this common garden study, we identified 15,072 different 16S rRNA operational taxonomic units (OTUs, 97% similarity), and 1027 fungal OTUs were identified from the ITS2 region.

### Rhizosphere microbiome response across the maize germplasm chronosequence

**Fig. 2:**
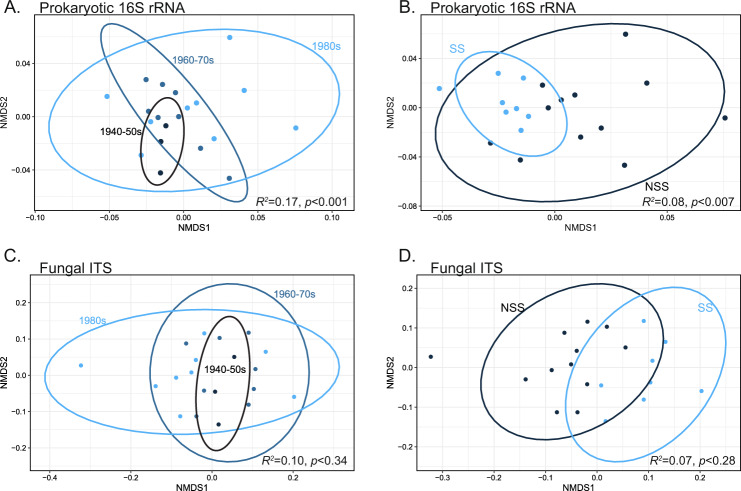
Changes in bacterial and fungal communities across a maize germplasm chronosequence. NMDS ordinations based on Bray–Curtis dissimilarity among prokaryotic 16S rRNA gene sequences (**A**, **B**) and fungal ITS gene sequences (**C**, **D**) obtained from rhizosphere microbiome samples, comparing prokaryotic and fungal community composition among maize genotypes representing different decades of germplasm development (**A**, **C**) or heterotic group (**B**, **D**). The two heterotic groups included in this study are non-stiff stalk (NSS) and stiff stalk (SS).

The decade of germplasm development, heterotic genetic group, and genotype all had a significant effect on rhizosphere microbiome composition. Plant genotype explained a significant amount of variance in the rhizosphere microbiome (PERMANOVA prokaryotic: *R*^2^ = 0.17, *p* < 0.001; fungal: *R*^2^ = 0.13, *p* < 0.001). When performing our analysis on the genotypic mean microbiome, we revealed that a decade of germplasm development explained 16.79% of the variance in the prokaryotic microbiome. In comparison, the heterotic group explained 8.1% of the variance (Fig. 2A, B, decade *p* < 0.01, heterotic *p* < 0.008, Table S4.1). Fungal microbiomes did not significantly respond to the germplasm chronosequence (*p* = 0.37) but differed among heterotic groups (*p* = 0.028) (Fig. 2C, D and Table S4.2).

### Response of nitrogen cycling functional groups to the germplasm chronosequence

From our analysis of nitrogen cycling functional genes, we observed 1498 *nifH* OTUs, 95 archaeal *amoA* OTUs, 200 bacterial *amoA* OTUs, 8632 *nirK* OTUs, 1186 *nirS* OTUs, 1068 *norB* OTUs, and 1864 *nosZ* OTUs. In response to the germplasm chronosequence, 3 of 7 nitrogen cycling genes showed changes in community membership, and 3 of 7 nitrogen cycling genes changed in copy number per ng of DNA (Table S5).

### Nitrogen fixation genes

**Fig. 3:**
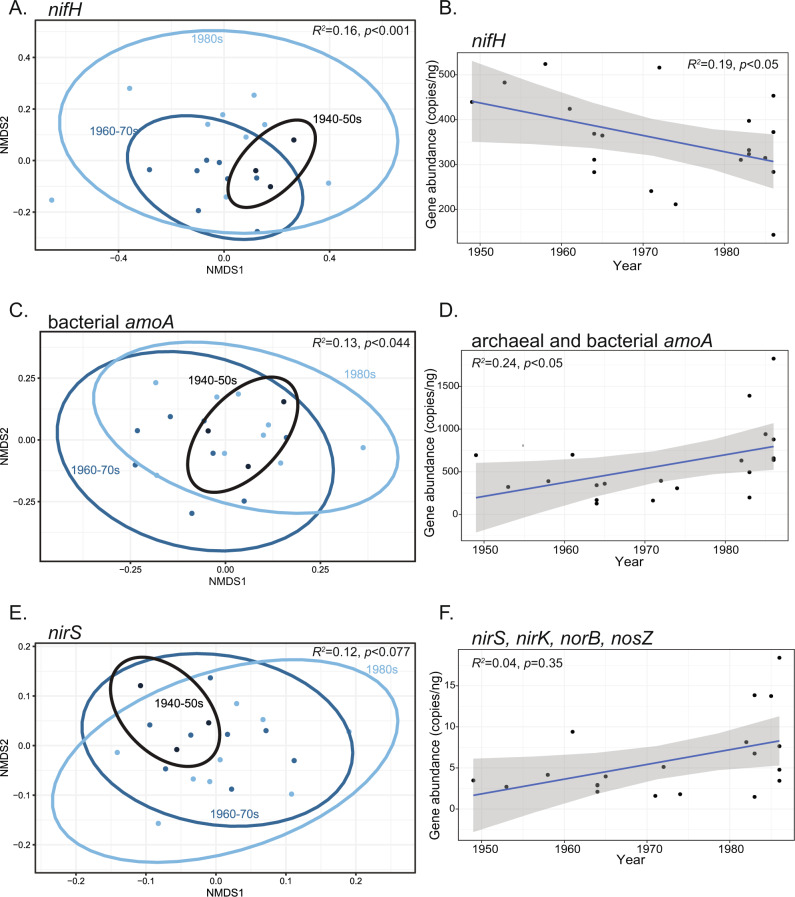
Changes in nitrogen cycling genes across a maize germplasm chronosequence. **A** NMDS ordination comparing maize rhizosphere *nifH* assemblages among decades of germplasm development. **B** Linear regression of *nifH* gene copy number across the maize germplasm chronosequence. **C** NMDS ordination comparing the composition of bacterial ammonia oxidizer assemblages among decades of germplasm development. **D** Linear regression of the sum of archaeal *amoA* and bacterial *amoA* gene abundance across the maize germplasm chronosequence. **E** NMDS ordination comparing assemblages of denitrifiers (based on *nirS* gene) across breeding decades. **F** Linear regression of the average qPCR abundance of denitrification genes (*nirS*, *nirK*, *norB*, *nosZ*) across the chronosequence. A complete list of statistical analyses of nitrogen cycling genes is presented in the supporting information: Tables S5–7 and Fig. S4.

There is a clear shift in the recruitment of nitrogen-fixing taxa across the germplasm chronosequence (Fig. 3A and Table S5). The composition of diazotrophs, detected through the nitrogenase *nifH* gene, was significantly impacted by the decade of germplasm development (*R*^2^ = 0.16, *p* < 0.001) and heterotic group (*R*^2^ = 0.13, *p* < 0.009, Fig. 3A, Table S6.1). The qPCR results also showed that the abundance of *nifH* in the microbiome significantly decreased across the germplasm chronosequence (*r* = −0.44, *p* < 0.05, Fig. 3B, linear model statistics in Table S7.1). These differences were detected even though the use of N fertilizer in our experiment abrogated any reliance on N fixation.

### Nitrification genes

The recruitment of nitrifiers (indicated by gene sequences for bacterial and archaeal ammonia monooxygenase—*amoA*) was shown to be significantly impacted by the germplasm chronosequence and heterotic group. We found a significant change in the composition of bacterial *amoA* genes (*R*^2^ = 0.13, *p* < 0.05, Fig. 3C, Table S6.2), but did not see a significant change in the abundance of bacterial *amoA* detected in response to the chronosequence (*p* = 0.14, Table S7.2). Archaeal nitrifiers showed no change in community composition over the chronosequence, but archaeal *amoA* genes did increase in abundance (*p* < 0.05, Fig. S4, Tables S6.3, S7.3). Total gene abundance of bacterial and archaeal *amoA* is significantly correlated with our chronosequence (*r* = 0.47, *p* < 0.05, Fig. 3D, Table S7.4).

### Denitrification genes

Several of the denitrification genes were significantly different among the chronosequence and heterotic groups (Figs. 3E, F, and S5, Tables S5, S6.4–7). Overall, changes in denitrifier communities had the weakest relationship to the chronosequence but rather were consistently driven by heterotic genetic group (Tables S6.4–7). Denitrifiers possessing the cytochrome *cd1*-type nitrite reductase, encoded by *nirS*, were the only denitrifier group showing altered composition in response to germplasm development (*p* = 0.07, Fig. 3E, Table S6.4). Only nitric oxide reductase, *norB*, gene abundance was correlated to time (*p* = 0.056, Tables S5, S6.7). All other denitrification genes lacked a significant correlation to the chronosequence (Tables S7.5–9). To summarize the gene abundance results, we averaged all the denitrification genes and regressed the mean abundance against the chronosequence (Fig. 3F). While this regression was not significant (*p* = 0.35, Table S7.9), gene abundance and chronosequence still had a positive relationship (*r* = 0.22).

### Identification and potential function of taxa that respond to the germplasm chronosequence

**Fig. 4:**
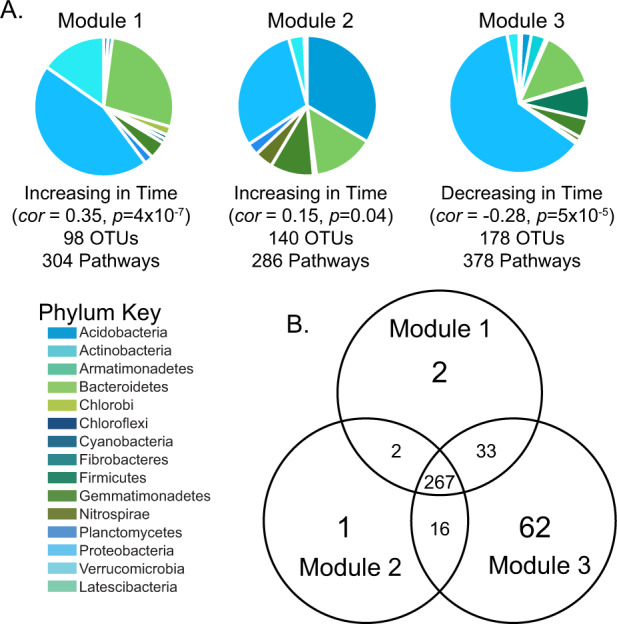
Taxa modules and predicted metabolic pathways for the maize rhizosphere microbiome, based on WGCNA and PICRUSt2 metabolic pathway predictions. **A** Modules (hierarchically clustered OTUs) that showed a significant correlation with the germplasm chronosequence. Module membership varies in size and taxonomic composition, grouped by phylum here. A list of dominant classes is presented in Table S9. **B** Venn diagram shows metabolic pathways shared across the three modules. Information on pathways is presented in Tables S10–12.

WGCNA [38] identified three unique sets of OTUs (modules) with a significant response to the germplasm chronosequence (Fig. 4A and Table S8). Modules 1 and 2 contained OTUs that were positively correlated to the decade of germplasm release, while Module 3 OTUs were negatively correlated with time. Module 1 contained 98 OTUs and was dominated by Proteobacteria. Module 2 contained 140 OTUs and was dominated by Actinobacteria. Module 3 contained 178 OTUs and was dominated by proteobacteria. Lists of dominant taxonomic classes from each module are presented in Table S9. Metagenomic functional predictions using PICRUSt2 were performed to predict the function of the taxa identified by WGCNA. Metagenomic functional predictions for the taxa in each module are presented in Figs. S6–7. PICRUSt2 predicted that the taxa in Module 1 had 304 pathways, Module 2 had 286 pathways, and Module 3 had 378 pathways. Among all modules, there was a high degree of shared predicted metabolic pathways (Fig. 4B). Module 1 and 2 (taxa increasing over the chronosequence) cumulatively contained only five unique pathways not present in Module 3. Module 3 (taxa decreasing in relative abundance across the chronosequence) had 62 unique pathways (Fig. 4B and Table S10). All modules revealed changes in the predicted abundance of pathways across the germplasm chronosequence: 83% of pathways in Module 1, 85% of pathways in Module 2, and 78% of pathways in Module 3 significantly changed across the germplasm chronosequence (Table S11). The rhizosphere microbiomes from germplasm developed during the 1940–50s were the most distinct in the predicted abundance of pathways compared to the lines released during the 1960s, 1970s, and 1980s. When comparing predicted microbial metabolic pathway differences among maize lines from the 1960–70s to the 1980s, little to no difference in abundance (0–0.002%) was found. This analysis allowed us to determine the pathways that showed the strongest response to our chronosequence (Table S12). Module 3 showed the most complex patterns of enrichment and depletion across our three decadal classifications (Fig. S6A). Module 1 and 2, while taxonomically distinct, appeared to be functionally redundant. Module 3 showed decreases across the chronosequence in pathways related to the degradation of organic nitrogen sources (Fig. S6B and Table S12). Across time all three modules were predicted to contain a greater number of gene pathways related to aerobic respiration and amino acid synthesis (Fig. S6C, D and Table S12). PICRUSt2 analysis was performed on correlated taxa modules. Description and analysis are presented in Supplemental materials.

### Genomic changes across the chronosequence

Supplementary analysis of the genomic variation within these maize lines was performed to assess whether we could gain mechanistic insight into the phenotypes causing our observed microbiome pattern. First, using TASSEL we scanned the haplotype diversity within the chronosequence population and found 87 large genetic regions (*p* < 0.05) showing evidence of undergoing recent selection events according to Tajima’s D statistic. Eighty-five of these regions were suggestive of a selective sweep and two of balancing selection (Supplemental materials Fig. S8). Next, we attempted to determine if changes in genomic variation corresponded to changes in our chronosequence timeline (Fig. S9). Using the maize HapMap, we first determined the population G matrix in TASSEL and then found the NMDS axes that explained most of the genetic variation (Figs. S2 and S9). These major NMDS axes were then regressed against our chronosequence timeline. Taken together this additional genetic analysis on the maize lines used in this study suggest that multiple alleles and a considerable amount of genetic variation were changed across this time period of maize development. Unfortunately, the design of this study lacks the power to determine the exact genes and traits driving this chronosequence microbiome pattern.

## Discussion

Plant rhizosphere microbiomes are, in part, shaped by plant genetics [10, 11]. Here we provide one of the few examples showing that selection (via breeding) on the plant genotype across a changing agronomic environment (i.e., increased synthetic N, increased plant density) drives changes in recruitment of the plant microbiome. We show that altered rhizosphere microbiome recruitment was reflected in functional genes for nitrification and nitrogen fixation and predicted metabolic pathways. Ultimately, these results suggest that breeding has altered the recruitment of soil microbiome and specific N-cycling functional groups in the maize rhizosphere.

Our results show a shift in rhizosphere prokaryotic microbiomes across a chronosequence of inbred maize lines, independent of broad genetic relatedness (heterotic group) (Fig. 2). These conclusions are based entirely on inbred maize lines and do not include hybrids. We decided to focus solely on inbred lines as hybrid maize genotypes exhibit a high degree of heterosis [18] and typically represent a highly genetically diverse combination of different heterotic pedigrees. This is important, as recent research has now established that this hybrid vigor can have considerable impacts on the assembly of the rhizosphere microbiome [43, 44]. Consequently, previous studies that included hybrids in their attempts to examine the effects of selection on maize through time unknowingly confounded heterosis and selection effects [45, 46]. Further research is needed to fully disentangle how hybridization shapes maize’s interactions with microbiomes and microbial functions related to agricultural sustainability.

The consequences of these microbiome changes extended to the composition and abundance of microbial genes associated with nitrogen cycling observed in the rhizosphere. We saw decreases in the abundance and changes in the composition of diazotrophs, indicated by *nifH*, in more recently developed germplasm (Fig. 2A, B). The increased usage of synthetic nitrogen fertilization through time has decreased maize’s reliance on microbial N provisioning. It is well established that the maintenance of belowground mutualistic N-fixation comes at a carbon cost [47], and alters aboveground carbon allocation across the plant [48]. In addition, altering nutrient availability modifies how a plant host assembles the microbiome [49]. In the breeding process, selection has been tuned to grain production [50] and weakened belowground carbon allocation [51]. Other findings in maize suggest that some landrace cultivars have a heightened ability to recruit associative diazotrophs [52]. Maize lines that host nitrogen-fixing bacteria produce specialized carbon-rich mucilage exudates to attract these microbes and gain substantial plant-available nitrogen from this interaction [47, 52]. Allocating carbon resources to the production of exudates comes at a cost to yield, and reliance on fixed N from diazotrophs is unnecessary under high nitrogen conditions [48], weakening any selection for maintenance of nutritional mutualisms that may have been present in ancestral maize lineages. Furthermore, under continuous high nitrogen fertilization, diazotrophs can evolve to become less efficient mutualistic nitrogen fixers [53]. A combination of these factors explains why maize’s recruitment of diazotrophs changes as a consequence of decades of crop selection.

Various functional genes related to denitrification and nitrification increased in abundance and changed in composition through the germplasm chronosequence (Fig. 2C–F and Table S5). While not all functional genes related to these processes responded to the chronosequence, especially those genes related to denitrification, there is still a clear pattern across time and germplasm selection. Changes in the abundance of N-cycling genes could be important in predicting losses of nitrogen and the production of GHGs from agroecosystems [54], as microorganisms that perform denitrification and nitrification can remove or alter the chemical structure and mobility of plant-available N [55]. Selective exudation of specialized metabolites from maize roots could be an explanation for shifts in the nitrifiers and denitrifiers across the germplasm represented in this study. For instance, different cereal grasses (sorghum, rice, wheat) have the ability to exude secondary phytochemical compounds that can suppress the metabolism of nitrifying organisms [56]. Here we hypothesize that a narrowing of germplasm diversity by inbreeding [12] could have eroded complex metabolic characteristics important for shaping interactions with nitrogen cycling microbial taxa [57]. Breeding of maize may have resulted in trait changes that influence how different cultivars recruit nitrogen cycling microbes. A growing body of research suggests that plants can drive the variability and activity of nitrifiers and denitrifiers in the soil ecosystem [56, 58–61]. Demonstrating that agroecosystem management and crop breeding altered the plant microbiome and potentially its functions suggest that plant-microbiome interactions are mutable—theoretically mutable enough that we can intentionally select for rhizosphere microbiome traits that contribute to nutrient retention, reduced GHG production, and improved soil health.

Plant species regulate microbial enzyme production and metagenomic capacity in the rhizosphere [62, 63]. Here we predicted changes in the microbial metagenome as a function of germplasm development. We found increases in the relative abundance of gene pathways related to amino acid biosynthesis and aerobic respiration. Gene pathways related to nitrogen substrate degradation decreased through the germplasm chronosequence (Figs. 3 and 4). These results imply that plants from earlier decades in this chronosequence support microbiomes that mineralize soil organic nitrogen, while later lines do the opposite. The rhizosphere microbiome of more recent germplasm is enriched for microbial taxa that have greater numbers of predicted metabolic pathways for respiration and amino acid synthesis. The predicted metagenome results suggest that the microbiome recruited by more modern germplasm is in a state of growth and biosynthesis. Coincidentally, these modern microbiomes are also predicted to have a lower capacity to mineralize free organic nitrogen sources; we hypothesize they are obtaining their nitrogen for biosynthesis from inorganic fertilizers, thereby potentially competing with the plant for nutrients instead of working mutualistically. The shift to aerobic respiration and simple sugar breakdown may indicate that the newer maize lines are recruiting copiotrophs [64]. These alterations to belowground predicted microbial metabolism could explain the observed yield gap between conventional and organic agroecosystems [65]. Maize lines that recruit fewer nutrient-mineralizing microbes may be compromised for the acquisition of nitrogen through organic nitrogen sources, resulting in lower yields. Currently, it is not well-established what type of soil metabolism would be ideal to meet our sustainability goals [6, 66]. However, these results indicate that we could breed germplasm to recruit microorganisms with traits that are aligned with soil management practices.

In conclusion, industrial breeding practices and agronomic management approaches have transformed maize’s interactions with its rhizosphere microbiome at a taxonomic and functional genomic level. These microbiome differences potentially alter nitrogen processing among plant cultivars and the movement of nitrogen in the agroecosystem as a whole. These changes likely occurred because of the combination of intense selection for aboveground traits and increased use of synthetic nitrogen fertilizers that reduced reliance on microbially-mediated nitrogen cycling processes. Modern agricultural practices have disrupted and accelerated the reactive nitrogen cycle. Maize has been a major contributor to this global disruption as it is one of the most farmed and fertilized crops in the world [67]. Alteration of plant microbiome function is indicated by recruitment of distinct assemblages of nitrogen-cycling taxa and predicted metabolic pathways in the rhizosphere microbiome of maize germplasm developed in different decades. Modern agricultural practices have accomplished the alteration of maize’s interaction with its root microbiome in the span of 50 years. Following these observations, the next steps would be to determine if the differences in microbiome recruitment are contributing to unsustainable outcomes in the agroecosystem and if unsustainable aspects of this microbiome recruitment are reversible. Approaching the microbiome and its functions as an extended phenotype of the plant genome will be a necessary step towards optimizing agricultural systems for sustainability.

## Supplementary information

Supplemental figures and tables

Supplemental tables S10-12
