# Supplementary material for: Maize germplasm chronosequence shows crop breeding history impacts recruitment of the rhizosphere microbiome
Source: ISME J. 2021 Mar 10;15(8):2454–64. doi: 10.1038/s41396-021-00923-z (PMC8319409; doi:10.1038/s41396-021-00923-z)
Supplement: Supplementary file 1 — Supplemental figures and tables [file 41396_2021_923_MOESM1_ESM.docx]

**Maize germplasm chronosequence shows crop breeding history impacts recruitment of the rhizosphere microbiome**

Alonso Favela^a^, Martin Bohn^b^, Angela Kent^a,c^

^a^Program of Ecology, Evolution, and Conservation Biology, University of Illinois at Urbana-Champaign, Urbana, IL 61801

^b^Department of Crop Sciences, University of Illinois at Urbana-Champaign, Urbana, IL 61801

^c^Department of Natural Resources and Environmental Sciences, University of Illinois at Urbana-Champaign, Urbana, IL 61801

**Supporting Information**

**Figure S1.** Visual abstract highlighting the major conclusion of the research. Circles represent the microbial taxa present in the rhizosphere after microbial recruitment and arrows represent microbial-plant interaction with nutrient environment. The goal of the figure is to highlight the changing nutrient environment that maize has experienced across different agricultural breeding settings during the 21^st^ century, and how this altered maize root systems and microbial interaction.

**Figure S2.** Shows the relationship between the heterotic groups and genetic relatedness of the lines used in the study based on HapMap2 genetic information from Panzea ([www.panzea.com](http://www.panzea.com)). **A.** Non-metric multidimensional scaling (NMDS) ordination based on genetic distance data of the genotypes used in the study. NonStiffStalk lines are colored as red. StiffStalk lines are colored as blue. This figure validates the usage of heterotic group as a proxy for genetic relatedness. **B.** Dendrogram showing how maize lines mostly cluster by heterotic group. NonStiffStalk lines are colored as red. StiffStalk lines are colored as blue. PHJ40 and Mo17 are the only exception to the heterotic group clustering.****

**Figure S3.** Workflow for Module analysis using WGCNA and PICRUSt2. Microbial communities were first run through a weighted correlation network analysis (WGCNA) to determine taxa responding to our consequence. These OTUs were then subset into modules and run through PICRSt2 where we were able to determine the predicted genes for the responding OTUs. BioCyc database was used to obtain functional additional metadata on genes and pathways of interest. This additional BioCyc metadata allowed to classify pathways by nuance categories such as super pathways, pathway inputs/outputs, and genes involved in pathway.

**Figure S4.** Ordinations displaying sequence composition for genes related to nitrification. **A**. archaeal *amoA* gene composition across breeding decade. **B**. archaeal *amoA* gene composition by heterotic groups. **C**. Denitrification *nirK* gene composition by heterotic groups. **D**. Dentification *nirK* gene composition across heterotic groups. **E.** *nosZ* gene composition by breeding decade **F.** *nosZ* composition by heterotic group **G.** *norB* composition by breeding decade **H.** *norB* composition by heterotic group

**

**Figure S5.** Abundance of nitrogen cycling genes changes (determined by qPCR ) across the germplasm chronosequence. **A**. Bacterial *amoA*. **B.** *nirK*. **C.** *nosZ*.


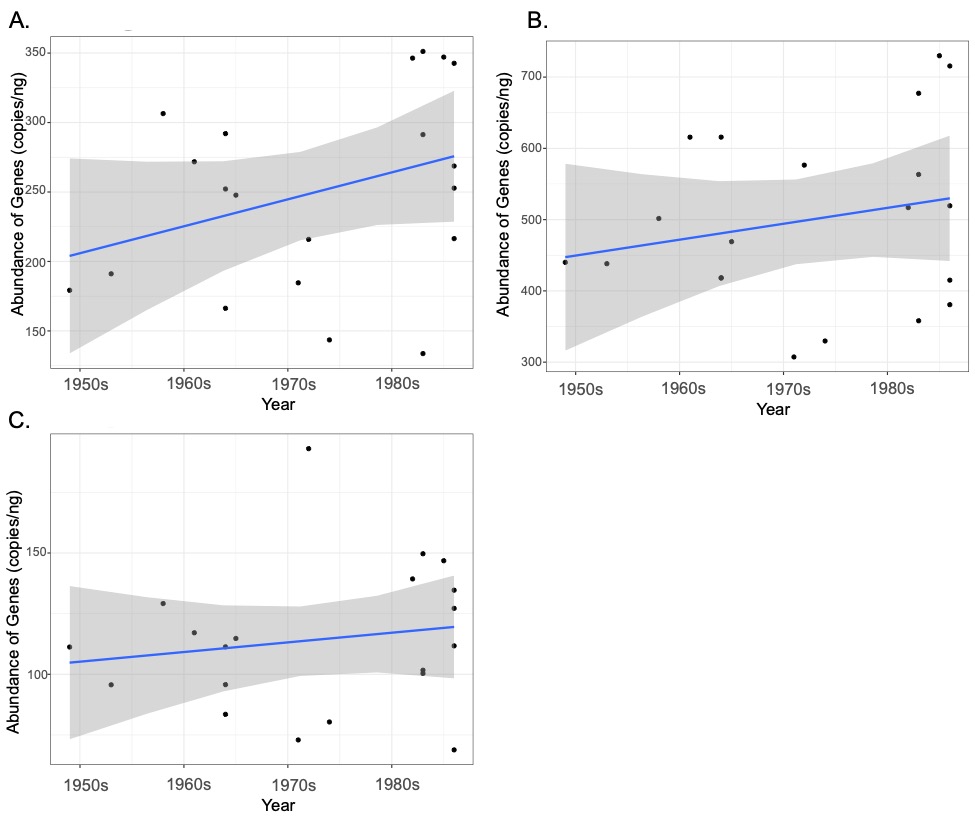


**Figure S6.** Changes in predicted pathways across chronosequence. **A.** Heatmap displaying predicted pathway differences of Module 3 across the germplasm chronosequence. Decade of germplasm development is present on the right y-axis. Colors on the top x-axis indicate bins representing broad functional categories for each pathway. Dendrogram on the left y-axis represents the similarity of the treatment categories. Darker colors signify higher Z-score abundances of pathways. Z-scores are relative based on pathway abundance. Heatmaps for other modules present in supplemental information Fig S6. **B**. Locally estimated scatter plot smoothing (LOESS) regression plot of the abundance for N degradation pathways over time. **C**. LOESS regression plot of the relative abundance of amino acid synthesis pathways over time. **D.** LOESS regression plot of the abundance of energy metabolism pathways across time.


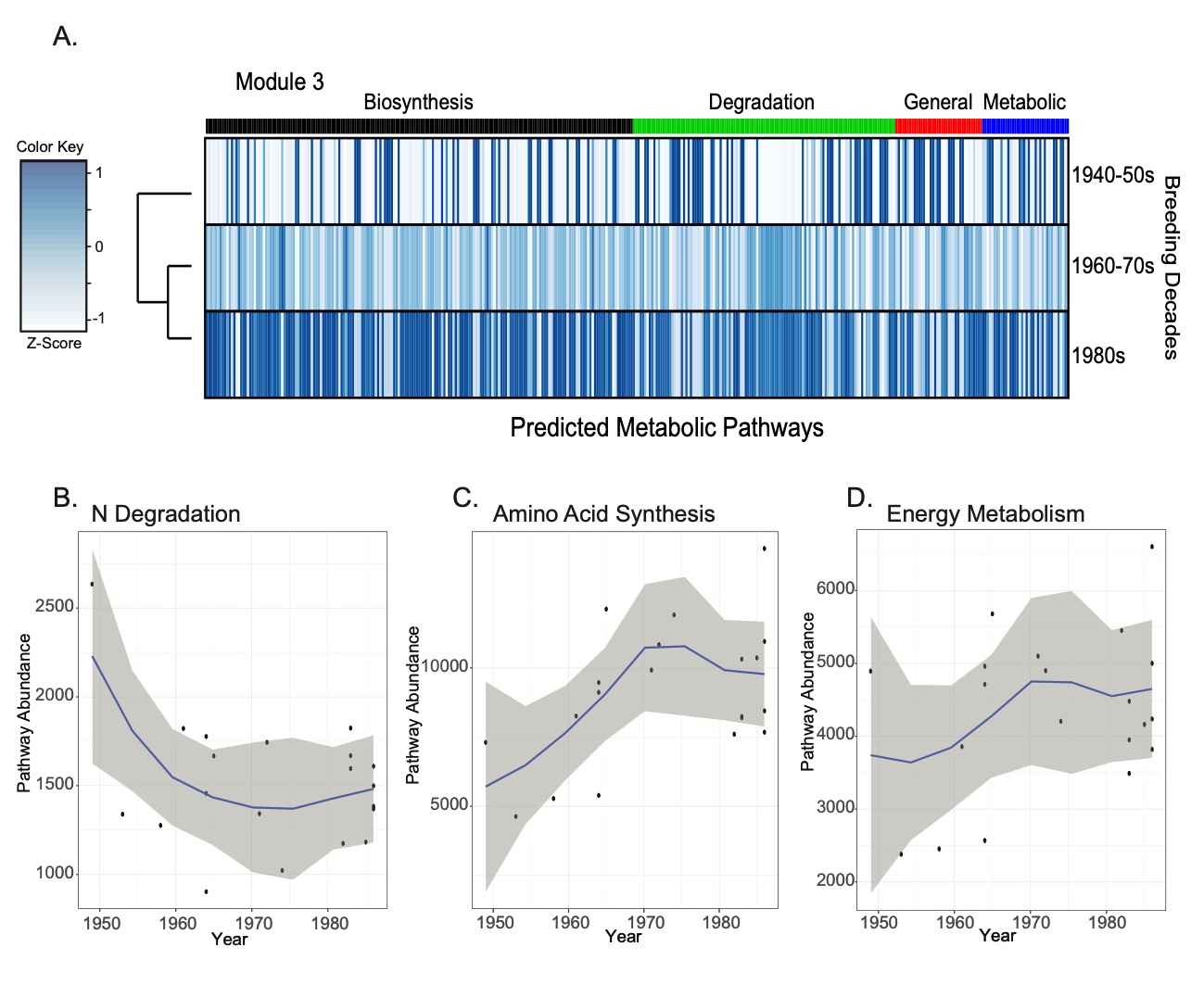


**Figure S7.** Heatmap results displaying predicted pathway differences across the taxonomic modules: **A.** module 1, **B.** module 2. Decade of germplasm release is indicated on the right y-axis. Colors on the top x-axis represent the broader functional category that the pathway is part of. Dendrogram on the left y-axis represents the similarity of the treatment categories. Darker colors signify higher Z-score abundances of pathways. Z-scores are relative based on pathway abundance.

**Figure S8.** Highlights the genomic changes that have occurred in these inbred lines over the chronosequence study. **A.** Non-metric multidimensional scaling (NMDS) ordination based on genetic distance data of the genotypes used in the study. Datapoint in this NMDS are the same as that in Fig S7A. labeled with the breeding decade color. **B.** Shows the Tajima’s D statistics across the HapMap of the lines included in this study. Tajima’s D is a basic genetic test used to understand whether genetic regions are evolving under selection pressures or neutrally/randomly within a population. This analysis revealed that 85 genomic regions experienced selective sweeps within our population, 2 under balancing selection, and the majority evolving randomly. The dotted lines in this figure are threshold lines which are 3 standard deviations away from the mean. The dots outside of this threshold compromise the 87 regions under suggested selection.

**Figure S9.** These figures make it clear that a significant amount of genetic variation is associated with the germplasm chronosequence. **Panels A and B** show a regression between the NMDS axes in Fig S8A and the year of germplasm development used in this study. Both figures show a significant amount of genomic variation is associated with the year of development. **A.** Highlights the regression between NMDS1 and year of development. **B.** Highlights the regression between NMDS2 and the year of development. These changes in genetic variation are likely what is driving microbiome patterns. **Panels** **C-F.** As NMDS dimensions are typically not used in regression analysis, we also ran a principal coordinates analysis (PCoA) to confirm our findings in A-B. The PCoA found 15 total axes in the HapMap genetic data. 10 of which were significantly correlated to with the year of development. Here we present the 4 significant correlations with the highest R values.

**Supporting information: Tables**

**Table S1.** Metadata of germplasm used in this study, sourced from the Mazie Genetic and Genomic Database (GDB).

| **Genotype** | **Heterotic group** | **Pedigree** | **Year** | **State** | **Decade** |
| --- | --- | --- | --- | --- | --- |
| B14 | StiffStalk | StiffStalkSyntheticC0 | 1953 | Iowa | 1940-50s |
| B37 | StiffStalk | StiffStalkSyntheticC0 | 1958 | Iowa | 1940-50s |
| OH43 | NonStiffStalk | Lancaster/Non-StiffStalk | 1949 | Iowa | 1940-50s |
| A619 | NonStiffStalk | Non-StiffStalk | 1961 | Minnesota | 1960-70s |
| A632 | StiffStalk | (Mt42 x B14)B14^3 | 1964 | Minnesota | 1960-70s |
| A634 | StiffStalk | (Mt42 x B14)B14@B14^2 | 1965 | Minnesota | 1960-70s |
| B73 | StiffStalk | StiffStalkSyntheticC5 | 1972 | Iowa | 1960-70s |
| LH1 | StiffStalk | (B37 x 644) B37 | 1974 | Iowa | 1960-70s |
| Mo17 | NonStiffStalk | Lancaster | 1964 | Missouri | 1960-70s |
| Pa91 | NonStiffStalk | Non-StiffStalk | 1971 | Pennsylvania | 1960-70s |
| W64A | NonStiffStalk | Non-StiffStalk | 1964 | Wisconsin | 1960-70s |
| LH123 | NonStiffStalk | Broadbase | 1983 | Iowa | 1980s |
| LH82 | NonStiffStalk | Broadbase/Minnesota | 1985 | Iowa | 1980s |
| PH207 | NonStiffStalk | Iodent | 1982 | Iowa | 1980s |
| PHG35 | NonStiffStalk | Oh07-Midland/Iodent | 1983 | Iowa | 1980s |
| PHG39 | StiffStalk | Stiff Stalk C0/Maiz Amargo | 1983 | Iowa | 1980s |
| PHG47 | NonStiffStalk | Oh43/Broadbase | 1986 | Iowa | 1980s |
| PHG84 | NonStiffStalk | Oh07-Midland/Broadbase | 1986 | Iowa | 1980s |
| PHJ40 | StiffStalk | StiffStalkC0/Broadbase | 1986 | Iowa | 1980s |
| PHZ51 | NonStiffStalk | Lancaster/Broadbase | 1986 | Iowa | 1980s |

**Table S2.** Primers used in amplicon sequencing and qPCR characterization of rhizosphere microbial community

| **Target** | **Encodes** | **Primer Name** | **Sequence** | **Reference** |
| --- | --- | --- | --- | --- |
| *16S rRNA* | Ribosomal RNA | 515F | 5'-GTGYCAGCMGCCGCGGTAA-3' | Fierer et al. 2011 |
| *16S rRNA* | Ribosomal RNA | 806R | 5'-GGACTACVSGGGTATCTAAT-3' | Fierer et al. 2011 |
| *ITS* | Internal Transcribed Spacer | ITS1F | 5'-TTCGTAGGTGAACCTGCGG-3' | White et al. 1990 |
| *ITS* | Internal Transcribed Spacer | ITS4R | 5'-TCCTCCGCTTATTGATATGC-3' | White et al. 1990 |
| *nifH* | Nitrogenase | PolF | 5'-TGCGAYCCSAARGCBGACTC-3' | Poly et al. 2001 |
| *nifH* | Nitrogenase | PolR | 5'-ATSGCCATCATYTCRCCGGA-3' | Poly et al. 2001 |
| bacterial *amoA* | Ammonia Monooxygenase | amoA-1F | 5'-GGGGTTTCTACTGGTGGT-3' | Oakley et al. 2005 |
| bacterial *amoA* | Ammonia Monooxygenase | amoA-2R | 5'-CCCCTCKGSAAAGCCTTCTTC-3' | Oakley et al. 2006 |
| archeal *amoA* | Ammonia Monooxygenase | CrenamoA23f | 5'-ATGGTCTGGCTWAGACG-3' | Francis et al. 2005 |
| archeal *amoA* | Ammonia Monooxygenase | CrenamoA616r | 5'-GCCATCCATCTGTATGTCCA-3' | Francis et al. 2005 |
| Typical *nosZ* | Nitrous oxide reductase | nosZ1F | 5'-WCSYTGTTCMTCGACAGCCAG-3' | Henry et al. 2006 |
| Typical *nosZ* | Nitrous oxide reductase | nosZ1R | 5'-ATGTCGATCARCTGVKCRTTYTC-3' | Henry et al. 2007 |
| *nirK* | Nitrite Reductase | nirK876 | 5'-ATYGGCGGVCAYGGCGA-3' | Henry et al. 2004 |
| *nirK* | Nitrite Reductase | nirK1040 | 5'-GCCTCGATCAGRTTRTGGTT-3' | Henry et al. 2004 |
| *nirS* | Nitrite Reductase | nirSCd3aF | 5'-AACGYSAAGGARACSGG-3' | Kandeler et al. 2006 |
| *nirS* | Nitrite Reductase | nirSR3cd | 5'-GASTTCGGRTGSGTCTTSAYGAA-3' | Kandeler et al. 2007 |
| *norB* | Nitric Oxide Reductase | cnorB2F | 5'-GACAAGNNNTACTGGTGGT-3' | Braker et al. 2003 |
| *norB* | Nitric Oxide Reductase | cnorB6R | 5'-GAANCCCCANACNCCNGC-3' | Braker et al. 2004 |

**Table S3.** Molecular sequencing raw reads generated from sequencing run, reads present after FASTX toolkit quality filter, and the rarefaction level of reads per sample for each gene used in this study.

| **Amplicon** | **Raw Reads** | **Quality Filtered Reads** | **Rarefaction Level** |
| --- | --- | --- | --- |
| **16S rRNA gene** | 45,616,533 | 39,079,559 | 34,000 |
| **fungal ITS** | 4,686,224 | 3,443,164 | 1,722 |
| **bacterial *amoA*** | 2,196,316 | 1,348,496 | 915 |
| **archaeal *amoA*** | 3,724,966 | 572,784 | 430 |
| ***nifH*** | 5,739,697 | 2,105,704 | 1353 |
| ***nirK*** | 13,544,866 | 4,972,273 | 999 |
| ***nirS*** | 3,375,608 | 1,102,622 | 165 |
| ***nosZ*** | 6,727,004 | 2,489,774 | 132 |
| ***norB*** | 2,317,818 | 560,953 | 100 |

**Table S4.** Permutational multivariate ANOVA model results at the genotypic level for 16S rRNA genes, and Fungal ITS*.* Standard model was run on all amplicon sequence data. Bray-Curtis distance was used to calculate dissimilarity between microbiomes. 999 permutations were used in analysis. All factors in the model were run as fixed effect: ***Microbial Community Matrix = Decade of release + Heterotic Group + Residuals.*** This model was used to all other nitrogen cycling genes compositional changes.

**Table S4.1** 16S rRNA: Genotypic means in PERMANOVA model

| **Terms** | **Df** | **SumsOfSqs** | **MeanSqs** | **F.Model** | ***R^2^*** | **Pr(>F)** | **Sig** |
| --- | --- | --- | --- | --- | --- | --- | --- |
| Decade | 2 | 0.07246 | 0.036231 | 1.7886 | 0.16792 | 0.001 | *** |
| Heterotic | 1 | 0.03496 | 0.034958 | 1.7257 | 0.08101 | 0.007 | ** |
| Residuals | 16 | 0.3241 | 0.020257 | 0.75107 |  |  |  |
| Total | 19 | 0.43152 | 1 |  |  |  |  |
| Signif. codes: 0 ‘***’ 0.001 ‘**’ 0.01 ‘*’ 0.05 ‘.’ 0.1 ‘ ’ 1 | | | | | | | |

**Table S4.2** Fungal ITS: Genotypic means in PERMANOVA model

| **Terms** | **Df** | **SumsOfSqs** | **MeanSqs** | **F.Model** | ***R^2^*** | **Pr(>F)** | **Sig** |
| --- | --- | --- | --- | --- | --- | --- | --- |
| Decade | 2 | 0.20778 | 0.103889 | 1.047 | 0.1073 | 0.34 |  |
| Heterotic | 1 | 0.14093 | 0.140935 | 1.4203 | 0.07278 | 0.028 | * |
| Residuals | 16 | 1.58763 | 0.099227 | 0.81991 |  |  |  |
| Total | 19 | 1.93635 | 1 |  |  |  |  |
| Signif. codes: 0 ‘***’ 0.001 ‘**’ 0.01 ‘*’ 0.05 ‘.’ 0.1 ‘ ’ 1 | | | | | | | |

**Table S5.** Functional genes that display significant patterns in composition and abundance across decades. Complete PERMANOVA and linear model outputs listed below. PERMANOVA Models: ***N-cycling Gene Matrix = Decade of release + Heterotic Group + Residuals.***

| **Process** | **Genes** | **Beta-Diversity Significance (*p*<0.05)** | **qPCR Abundance Significance in Relation to Time (*p*<0.05 )** |
| --- | --- | --- | --- |
| Nitrogen Fixation | *nifH* | Significant * | Significant * |
| Nitrification | Bacterial *amoA* | Significant * | Non-Significant |
| Nitrification | Archeal *amoA* | Non-Significant | Significant * |
| Denitrification | *nirK* | Non-Significant | Non-Significant |
| Denitrification | *nirS* | Near-Significant^•^ | Non-Significant |
| Denitrification | *norB* | Non-Significant | Significant * |
| Denitrification | *nosZ* | Non-Significant | Non-Significant |

**Table S6.** Nitrogen cycling functional groups PERMANOVA result. Model: ***N-cycling Gene Matrix = Decade of release + Heterotic Group + Residuals.*** Includes *nifH,* Bacterial *amoA,* Archaeal *amoA, nirS, nirK, nosZ, norB.*

**Table S6.1** *nifH* gene: Genotypic Means PERMANOVA Model

| **Terms** | **Df** | **SumsOfSqs** | **MeanSqs** | **F.Model** | ***R^2^*** | **Pr(>F)** | **Sig** |
| --- | --- | --- | --- | --- | --- | --- | --- |
| Decade | 2 | 0.07853 | 0.039263 | 1.7024 | 0.16123 | 0.001 | *** |
| Heterotic | 1 | 0.03952 | 0.039515 | 1.7133 | 0.08113 | 0.003 | ** |
| Residuals | 16 | 0.36902 | 0.023064 | 0.75764 |  |  |  |
| Total | 19 | 0.48706 | 1 |  |  |  |  |
| Signif. codes: 0 ‘***’ 0.001 ‘**’ 0.01 ‘*’ 0.05 ‘.’ 0.1 ‘ ’ 1 | | | | | | | |

**Table S6.2** Bacterial *amoA* gene: Genotypic Means PERMANOVA Model

| **Terms** | **Df** | **SumsOfSqs** | **MeanSqs** | **F.Model** | ***R^2^*** | **Pr(>F)** | **Sig** |
| --- | --- | --- | --- | --- | --- | --- | --- |
| Decade | 2 | 0.11113 | 0.055564 | 1.4361 | 0.1376 | 0.044 | * |
| Heterotic | 1 | 0.07745 | 0.077454 | 2.0019 | 0.0959 | 0.008 | ** |
| Residuals | 16 | 0.61905 | 0.038691 | 0.7665 |  |  |  |
| Total | 19 | 0.80763 | 1 |  |  |  |  |
| Signif. codes: 0 ‘***’ 0.001 ‘**’ 0.01 ‘*’ 0.05 ‘.’ 0.1 ‘ ’ 1 | | | | | | | |

| **Table S6.3** Archaeal *amoA* gene: Genotypic Means PERMANOVA Model | | | | | | | |  |
| --- | --- | --- | --- | --- | --- | --- | --- | --- |
| **Terms** | **Df** | **SumsOfSqs** | **MeanSqs** | **F.Model** | ***R^2^*** | **Pr(>F)** | **Sig** | |
| Decade | 2 | 0.020158 | 0.010079 | 0.77578 | 0.07965 | 0.755 |  | |
| Heterotic | 1 | 0.02505 | 0.02505 | 1.92813 | 0.09898 | 0.054 | . | |
| Residuals | 16 | 0.207874 | 0.012992 | 0.82137 |  |  |  | |
| Total | 19 | 0.253082 | 1 |  |  |  |  | |
| Signif. codes: 0 ‘***’ 0.001 ‘**’ 0.01 ‘*’ 0.05 ‘.’ 0.1 ‘ ’ 1 | | | | | | | | |

**Table S6.4** *nirS* gene*:* Genotypic Means PERMANOVA Model

| \| **Terms** \| **Df** \| **SumsOfSqs** \| **MeanSqs** \| **F.Model** \| ***R^2^*** \| **Pr(>F)** \| **Sig** \| \| --- \| --- \| --- \| --- \| --- \| --- \| --- \| --- \| \| Decade \| 2 \| 0.33868 \| 0.16934 \| 1.2166 \| 0.12427 \| 0.077 \| . \| \| Heterotic \| 1 \| 0.15965 \| 0.15965 \| 1.1469 \| 0.05858 \| 0.213 \|  \| \| Residuals \| 16 \| 2.2271 \| 0.13919 \| 0.81716 \|  \|  \|  \| \| Total \| 19 \| 2.72543 \| 1 \|  \|  \|  \|  \| \| Signif. codes: 0 ‘***’ 0.001 ‘**’ 0.01 ‘*’ 0.05 ‘.’ 0.1 ‘ ’ 1 \| \| \| \| \| \| \| \|   **Table S6.5** *nirK* gene: Genotypic Means PERMANOVA Model | | | | | | | | |
| --- | --- | --- | --- | --- | --- | --- | --- | --- | --- | --- | --- | --- | --- | --- | --- | --- | --- | --- | --- | --- | --- | --- | --- | --- | --- | --- | --- | --- | --- | --- | --- | --- | --- | --- | --- | --- | --- | --- | --- | --- | --- | --- | --- | --- | --- | --- | --- | --- | --- | --- | --- | --- | --- | --- | --- | --- |
| **Terms** | **Df** | **SumsOfSqs** | **MeanSqs** | **F.Model** | ***R^2^*** | **Pr(>F)** | **Sig** |  |
| Decade | 2 | 0.4336 | 0.2168 | 1.0147 | 0.10599 | 0.365 |  |  |
| Heterotic | 1 | 0.2387 | 0.23873 | 1.1173 | 0.05835 | 0.059 | . |  |
| Residuals | 16 | 3.4188 | 0.21367 | 0.83566 |  |  |  |  |
| Total | 19 | 4.0911 | 1 |  |  |  |  |  |
| Signif. codes: 0 ‘***’ 0.001 ‘**’ 0.01 ‘*’ 0.05 ‘.’ 0.1 ‘ ’ 1 | | | | | | | |  |

**Table S6.6** *nosZ* gene: Genotypic Means PERMANOVA Model

| **Terms** | **Df** | **SumsOfSqs** | **MeanSqs** | **F.Model** | ***R^2^*** | **Pr(>F)** | **Sig** |
| --- | --- | --- | --- | --- | --- | --- | --- |
| Decade | 2 | 0.5213 | 0.26066 | 0.93175 | 0.0975 | 0.841 |  |
| Heterotic | 1 | 0.3497 | 0.34968 | 1.24996 | 0.0654 | 0.017 | * |
| Residuals | 16 | 4.4761 | 0.27975 | 0.83711 |  |  |  |
| Total | 19 | 5.3471 | 1 |  |  |  |  |
| Signif. codes: 0 ‘***’ 0.001 ‘**’ 0.01 ‘*’ 0.05 ‘.’ 0.1 ‘ ’ 1 | | | | | | | |

**Table S6.7** *norB* gene: Genotypic Means PERMANOVA Model

| **Terms** | **Df** | **SumsOfSqs** | **MeanSqs** | **F.Model** | ***R^2^*** | **Pr(>F)** | **Sig** |
| --- | --- | --- | --- | --- | --- | --- | --- |
| Decade | 2 | 0.6115 | 0.30577 | 1.0037 | 0.10355 | 0.445 |  |
| Heterotic | 1 | 0.4202 | 0.42021 | 1.3794 | 0.07115 | 0.005 | ** |
| Residuals | 16 | 4.8741 | 0.30463 | 0.8253 |  |  |  |
| Total | 19 | 5.9059 | 1 |  |  |  |  |
| Signif. codes: 0 ‘***’ 0.001 ‘**’ 0.01 ‘*’ 0.05 ‘.’ 0.1 ‘ ’ 1 | | | | | | | |

**Table S7.** Mixed effect models comparing qPCR of functional genes and year of germplasm release while corrected for the genetic relatedness between lines used in the study. Year of release was run as a fixed effect, genetic relatedness between lines was run as a random effect. Wald tests performed on statistical models to calculate significance. Statistical models were run in ‘asreml-r’.

**Table S7.1** *nifH* gene:

asreml(fixed=*nifH qPCR*~1+Year, Random=vm(Genotype, inverseGmatrix)

| Terms | Df | Sum of Sq | Wald Statistic | Pr(Chisq) |  |
| --- | --- | --- | --- | --- | --- |
| (Intercept) | 1 | 14097925 | 860.68 | <2e-16 | *** |
| Year | 1 | 66351 | 4.05 | 0.04415 | * |
| residual (MS) |  | 16380 |  |  |  |
| Signif. codes: 0 ‘***’ 0.001 ‘**’ 0.01 ‘*’ 0.05 ‘.’ 0.1 ‘ ’ 1 | | | | | |
| Variance components of GMatrix | | | | | |
|  | component | std.error | z.ratio | bound | %ch |
| vm(Genotype, InvGMatrix**)** | 22757.97 | 10339.839 | 2.200998 | P | 0.2 |
| units!R | 16380.04 | 5723.233 | 2.862025 | P | 0 |

**Table S7.2** Bacterial *amoA* gene:

asreml(fixed=*BamoAqPCR*~1+Year, Random=vm(Genotype, inverseGmatrix)

| Terms | Df | | Sum of Sq | | Wald Statistic | | Pr(Chisq) | |  | |  |
| --- | --- | --- | --- | --- | --- | --- | --- | --- | --- | --- | --- |
| (Intercept) | 1 | | 1548270 | | 332.22 | | <2e-16 | | *** | |  |
| Year | 1 | | 10159 | | 2.18 | | 0.1398 | |  | |  |
| residual (MS) |  | | 4660 | |  | |  | |  | |  |
| Signif. codes: 0 ‘***’ 0.001 ‘**’ 0.01 ‘*’ 0.05 ‘.’ 0.1 ‘ ’ 1 | | | | | | | | | | |  |
| Variance components of GMatrix | | | | | | | | | | |  |
|  | | component | | std.error | | z.ratio | | bound | | %ch | |
| vm(Genotype, InvGMatrix**)** | | 22757.97 | | 10339.839 | | 2.200998 | | P | | 0.2 | |
| units!R | | 16380.04 | | 5723.233 | | 2.862025 | | P | | 0 | |

**Table S7.3** Archaeal *amoA* gene:

asreml(fixed=*ArchamoA qPCR*~1+Year, Random=vm(Genotype, inverseGmatrix)

| Terms | Df | Sum of Sq | Wald Statistic | Pr(Chisq) |  |
| --- | --- | --- | --- | --- | --- |
| (Intercept) | 1 | 15789973 | 97.925 | <2e-16 | *** |
| Year | 1 | 714521 | 4.431 | 0.03529 | * |
| residual (MS) |  | 161245 |  |  |  |
| Signif. codes: 0 ‘***’ 0.001 ‘**’ 0.01 ‘*’ 0.05 ‘.’ 0.1 ‘ ’ 1 | | | | | |

Variance components of GMatrix

|  | component | std.error | z.ratio | bound | %ch |
| --- | --- | --- | --- | --- | --- |
| vm(Genotype, InvGMatrix**)** | 204081.8 | 109931.36 | 1.856448 | P | 0.2 |
| units!R | 161244.8 | 55997.15 | 2.879517 | P | 0 |

**Table S7.4** Sum of archaeal and bacterial *amoA* genes:

asreml(fixed=*AamoA qPCR+BamoA qPCR*~1+Year, Random=vm(Genotype, inverseGmatrix)

| Terms | Df | Sum of Sq | Wald Statistic | Pr(Chisq) |  |
| --- | --- | --- | --- | --- | --- |
| (Intercept) | 1 | 27023187 | 138.268 | 2.00E-16 | *** |
| Year | 1 | 895278 | 4.581 | 0.03233 | * |
| residual (MS) |  | 195441 |  |  |  |
| Signif. codes: 0 ‘***’ 0.001 ‘**’ 0.01 ‘*’ 0.05 ‘.’ 0.1 ‘ ’ 1 | | | | | |

Variance components of GMatrix

|  | component | std.error | z.ratio | bound | %ch |
| --- | --- | --- | --- | --- | --- |
| vm(Genotype, InvGMatrix**)** | 225302.6 | 143531.36 | 1.56971 | P | 0.2 |
| units!R | 195440.6 | 67901.14 | 2.878311 | P | 0 |

**Table S7.5** *nirK* gene:

asreml(fixed=*nirK qPCR*~1+Year, Random=vm(Genotype, inverseGmatrix)

| Terms | Df | Sum of Sq | Wald Statistic | Pr(Chisq) |  |
| --- | --- | --- | --- | --- | --- |
| (Intercept) | 1 | 5796044 | 357.98 | <2e-16 | *** |
| Year | 1 | 12693 | 0.78 | 0.3759 |  |
| residual (MS) |  | 16191 |  |  |  |
| Signif. codes: 0 ‘***’ 0.001 ‘**’ 0.01 ‘*’ 0.05 ‘.’ 0.1 ‘ ’ 1 | | | | | |
| Variance components of GMatrix | | | | | |
|  | component | std.error | z.ratio | bound | %ch |
| vm(Genotype, InvGMatrix**)** | 4915.47 | 17857.128 | 0.2752666 | P | 23.5 |
| units!R | 16191.05 | 5676.988 | 2.8520498 | P | 0 |

**Table S7.6** *nirS* gene:

Here asreml-r model did not converge so we used a general genotypic means in linear Model

| Terms | Estimate | Std. | Error | t | value | Pr(>\|t\|) |
| --- | --- | --- | --- | --- | --- | --- |
| (Intercept) | -198.9049 | 528.6676 | -0.376 | 0.711 |  |  |
| qPCR vs Year | 0.1135 | 0.268 | 0.424 | 0.677 |  |  |
| Signif. codes: 0 ‘***’ 0.001 ‘**’ 0.01 ‘*’ 0.05 ‘.’ 0.1 ‘ ’ 1  **Table S7.7** *norB* gene:  asreml(fixed=*norB qPCR*~1+Year, Random=vm(Genotype, inverseGmatrix)   \| Terms \| Df \| Sum of Sq \| Wald Statistic \| Pr(Chisq) \|  \| \| --- \| --- \| --- \| --- \| --- \| --- \| \| (Intercept) \| 1 \| 883.96 \| 46.404 \| 9.62E-12 \| *** \| \| Year \| 1 \| 87.37 \| 4.586 \| 0.03223 \| * \| \| residual (MS) \|  \| 19.05 \|  \|  \|  \| \| Signif. codes: 0 ‘***’ 0.001 ‘**’ 0.01 ‘*’ 0.05 ‘.’ 0.1 ‘ ’ 1 \| \| \| \| \| \| | | | | | | |

Variance components of GMatrix

|  | component | std.error | z.ratio | bound | %ch |
| --- | --- | --- | --- | --- | --- |
| vm(Genotype, InvGMatrix**)** | 10.45639 | 17.18785 | 0.6083591 | P | 19.3 |
| units!R | 19.04921 | 6.68549 | 2.8493364 | P | 0 |

**Table S7.8** *nosZ* gene:

asreml(fixed=*nosZ qPCR*~1+Year, Random=vm(Genotype, inverseGmatrix)

| Terms | Df | Sum of Sq | Wald Statistic | Pr(Chisq) |  |
| --- | --- | --- | --- | --- | --- |
| (Intercept) | 1 | 6530366 | 6340.7 | <2e-16 | *** |
| Year | 1 | 154 | 0.1 | 0.6988 |  |
| residual (MS) |  | 1030 |  |  |  |
| Signif. codes: 0 ‘***’ 0.001 ‘**’ 0.01 ‘*’ 0.05 ‘.’ 0.1 ‘ ’ 1 | | | | | |

Variance components of GMatrix

|  | component | std.error | z.ratio | bound | %ch |
| --- | --- | --- | --- | --- | --- |
| vm(Genotype, InvGMatrix**)** | 142.2504 | 6049.3431 | 0.02351502 | ? | 93.7 |
| units!R | 1029.9048 | 737.2589 | 1.39693766 | P | 0 |

**Table S7.9** Average of denitrification genes:

asreml(fixed=*nosZ+norB+nirK+nirS)/4*~1+Year, Random=vm(Genotype, inverseGmatrix)

| Terms | Df | Sum of Sq | Wald Statistic | Pr(Chisq) |  |
| --- | --- | --- | --- | --- | --- |
| (Intercept) | 1 | 706604 | 410.01 | <2e-16 | *** |
| Year | 1 | 1178 | 0.68 | 0.4084 |  |
| residual (MS) |  | 1723 |  |  |  |
| Signif. codes: 0 ‘***’ 0.001 ‘**’ 0.01 ‘*’ 0.05 ‘.’ 0.1 ‘ ’ 1 | | | | | |

Variance components of GMatrix

|  | component | std.error | z.ratio | bound | %ch |
| --- | --- | --- | --- | --- | --- |
| vm(Genotype, InvGMatrix**)** | 967.5626 | 1614.2786 | 0.5993777 | P | 19.8 |
| units!R | 1723.3808 | 607.6169 | 2.836295 | P | 0 |

**Table S8.** Statistically significant modules and all WGCNA Results. Supplemental table with full correlations between microbial modules is available for downloaded.

| **Module** | **Trait** | **Cor** | **pval** | **Sig** |
| --- | --- | --- | --- | --- |
| Module 3 | Year | -0.2816814 | 5.33E-05 | TRUE |
| Module 2 | Year | 0.1454025 | 0.03994247 | TRUE |
| Module 1 | Year | 0.34856317 | 4.24E-07 | TRUE |

**Table S9.** Dominant taxonomic classes present in modules identified from the WGCNA.

| Module 1 | | Module 2 | | Module 3 | |
| --- | --- | --- | --- | --- | --- |
| Class | Count | Class | Count | Class | Count |
| Betaproteobacteria | 16 | Subgroup 6 | 23 | Alphaproteobacteria | 58 |
| Alphaproteobacteria | 16 | Sphingobacteriia | 17 | Betaproteobacteria | 29 |
| Opitutae | 11 | Alphaproteobacteria | 13 | Gammaproteobacteria | 19 |
| Sphingobacteriia | 10 | Betaproteobacteria | 13 | Sphingobacteriia | 16 |
| Cytophagia | 14 | Gammaproteobacteria | 11 | Clostridia | 10 |
| Fibrobacteria | 1 | Blastocatellia | 10 | Gemmatimonadetes | 7 |
| Deltaproteobacteria | 7 | Gemmatimonadetes | 9 | Actinobacteria | 5 |
| Solibacteres | 1 | Holophagae | 8 | Deltaproteobacteria | 4 |
| Gammaproteobacteria | 5 | Nitrospira | 6 | Negativicutes | 4 |
| Melainabacteria | 2 | Deltaproteobacteria | 5 | Cytophagia | 3 |
| Flavobacteriia | 3 | S0134 terrestrial group | 4 | Verrucomicrobiae | 3 |
| Chlorobia | 2 | Cytophagia | 3 | Bacteroidia | 2 |
| Gemmatimonadetes | 4 | OPB35 soil group | 2 | Nitrospira | 2 |
| OPB35 soil group | 3 | Subgroup 5 | 2 | Flavobacteriia | 2 |
| Verrucomicrobiae | 1 | Phycisphaerae | 2 | Holophagae | 2 |
| vadinHA49 | 2 | Solibacteres | 2 | Subgroup 6 | 2 |
|  |  | Verrucomicrobiae | 2 | Fimbriimonadia | 1 |
|  |  | Spartobacteria | 1 | Longimicrobia | 1 |
|  |  | Subgroup 17 | 1 | Planctomycetacia | 1 |
|  |  | Anaerolineae | 1 | Opitutae | 1 |
|  |  | Longimicrobia | 1 | Fibrobacteria | 1 |
|  |  | OM190 | 1 | WCHB1-32 | 1 |
|  |  | Subgroup 11 | 1 | Proteobacteria | 1 |
|  |  | Planctomycetacia | 1 | Acidimicrobiia | 1 |
|  |  | uncultured bacterium | 1 | OPB35 soil group | 1 |

**Table S10.** Displays the list of pathways predicted for each of the taxonomic modules from PICRUSt2 analysis. Additional meta-information of the pathways is present in supplemental excel tables. Pathway names are in MetaCyc formatting. Supplemental excel table available for download.

**Table S11.** Number of pathways significantly different pathways across decade of germplasm development from SIMPER comparison. Table S14 contains full pathway names. Supplemental excel table available for download.

**Table S12.** SIMPER results displaying pathways that are significantly different across the germplasm chronosequence. The mean differences values signify the differences in predicted gene abundance in pathway between the decades of comparisons. All pathways listed were shown to be statistically significant (*p<*0.05), pathways are organized by the degree of mean difference between the groups. Supplemental excel table available for download.

**Table S12.1** Results from Module 1 comparison between germplasm developed in the 1940-50s to the 1960-70s.

**Table S12.2** Results from Module 1 comparison between germplasm developed in the 1940-50s to the 1980s.

**Table S12.3** Results of Module 2 comparison between germplasm developed in the 1940-50s to the 1960-70s.

**Table S12.4** Results of Module 2 comparison between germplasm developed in the 40-50s to the 80s.

**Table S12.5** Results of Module 3 comparison between germplasm developed in the 1940-50s to the 1960-70s.

**Table S12.6** Results of Module 3 comparison between germplasm developed in the 1940-50s to the 1980s.
